# Evaluating cognitive and motivational accounts of greater reinforcement effects among children with attention-deficit/hyperactivity disorder

**DOI:** 10.1186/s12993-015-0065-9

**Published:** 2015-04-29

**Authors:** Whitney D Fosco, Larry W Hawk, Keri S Rosch, Michelle G Bubnik

**Affiliations:** Departments of Psychology, University at Buffalo, SUNY, 206 Park Hall, Box 604110, Buffalo, NY USA; Center for Children and Families, University at Buffalo, SUNY, 206 Park Hall, Box 604110, Buffalo, NY USA; Kennedy Krieger Institute, Baltimore, MD USA

**Keywords:** Attention-deficit/hyperactivity disorder, ADHD, Reinforcement, Working memory, Inhibitory control, Attention, Cognition, Sensitivity to reward

## Abstract

**Background:**

Attention Deficit/Hyperactivity Disorder is associated with cognitive deficits and dysregulated motivation. Reinforcement improves cognitive performance, often to a greater degree among children with ADHD compared to typically-developing controls. The current study tests the degree to which cognitive (individual differences in baseline cognition) and/or motivational (individual differences in Sensitivity to Reward; SR) processes can account for diagnostic group differences in reinforcement effects.

**Methods:**

Participants were 58 children (25 ADHD, 33 control) ages 9-12. Children completed measures of inhibitory control (Stop Signal Task), working memory (n-back), and sustained attention (Continuous Performance Task) during a baseline week and again one week later under reinforcement and no-reinforcement conditions; composites were computed across cognitive domains. Parent-and child-reported trait SR (SPSRQ; BIS/BAS) were combined to index a child’s response towards appetitive, rewarding stimuli.

**Results:**

In separate analyses, diagnostic group, individual differences in baseline cognition, and individual differences in SR all moderated the impact of reinforcement on cognition. When considered together, the Diagnostic Group × Reinforcement and Baseline Cognition × Reinforcement interactions both remained robust. In contrast, neither the Diagnostic Group × Reinforcement nor the SR × Reinforcement interactions accounted for unique variance when evaluated together.

**Conclusions:**

Both baseline cognition and trait SR predict reinforcement effects on cognition, but only SR shares significant variance with diagnostic group. These results suggest that ADHD children’s greater response to reinforcement on cognition is strongly related to their heightened trait sensitivity to rewarding stimuli, consistent with motivational models of ADHD.

Attention Deficit/Hyperactivity Disorder (ADHD) is diagnosed based on cross-situational patterns of inattentive and/or hyperactive/impulsive symptoms [1] and is associated with impairment in multiple cognitive domains [2-4] and motivational processes [5-7]. Recent work in neuroscience suggests that the two systems function interactively, with motivational parameters modulating cognitive performance [8-10].

A growing number of studies have examined the impact of reinforcement on laboratory measures of cognition in children with and without ADHD, with a key prediction being that the impact of reinforcement should be greater in ADHD than controls (see [11]). Though not all studies observe this pattern [12,13], there is growing evidence that continuous reinforcement for cognitive performance is more beneficial for children with ADHD than controls [14-16]. Attempts to explain Diagnostic Group × Reinforcement interactions in the ADHD literature have generally focused on motivational or cognitive deficits [17]. A motivational account would suggest that the differential group effect of reinforcement is caused by dysfunctional reinforcement processing among ADHD children [18,19]. Indeed, research utilizing self- or parent-report measures suggests that children with ADHD are more sensitive to rewards than their typically-developing peers [20]. Sensitivity to Reward (SR) is based on Gray’s Behavioral Approach System (BAS), a motivational system hypothesized to activate in the presence of rewards, resulting in approach behavior [21]. Individual differences in SR are predictive of externalizing behavior [22] and physiological responses to rewards [23,24], suggesting that it may predict who will benefit most from reinforcement, regardless of diagnostic status.

Differential group responses to reinforcement may also be a function of differences in baseline cognition [9,16]. Children with ADHD may show more robust responses to reinforcement due to their poor performance in no-reinforcement task conditions, which allows greater room for improvement; conversely, control children typically perform well on even no-reinforcement versions of tasks, and ceiling effects among this group may attenuate any possible effect of reinforcement. However, in order to examine the extent to which baseline differences contribute to Diagnostic Group × Reinforcement interactions, a true “baseline” must be obtained. The single-session comparison of reinforcement and no-reinforcement that characterizes most ADHD work in the area [16] may be influenced by the overall reinforcement context of the session, learning during the reinforcement condition, or even frustrative non-reward following reinforcement delivery [25]. To provide separate indices of baseline cognition and reinforcement effects on cognition, we tested children in standard (baseline) cognitive paradigms during an initial visit and then under reinforcement and no-reinforcement conditions in a subsequent visit.

The present study is the first to evaluate the degree to which individual differences in SR and individual differences in baseline cognitive performance contribute to greater effects of reinforcement on cognitive performance in children with ADHD than in typical controls. To do this, we combined data from reports showing Diagnostic Group × Reinforcement interactions on tasks of inhibitory control [26], working memory [15], and sustained attention [27], as these processes are considered central in ADHD. We predict the following pattern of findings: 1) when examined independently, diagnostic group, baseline cognition (measured one week prior to the reinforcement manipulation version of tasks), and parent- and child-composite SR will each moderate the effect of reinforcement on cognition; we expect ADHD children, those with low baseline cognition, and those with high SR to show more robust reinforcement effects; 2) when evaluating the extent to which group differences in reinforcement effects are due to shared variance with baseline cognition and/or SR, we predict that the Baseline Cognition × Reinforcement and SR × Reinforcement interactions will account for unique variance but that the Diagnostic Group × Reinforcement interaction will not. That is, we expect individual differences in both cognition and motivation to share significant variance with what we consider “ADHD,” and including these dimensional processes thought central to the disorder will render diagnostic status non-significant in predicting reinforcement effects on cognition.

## Method

### Participants

Fifty-eight children, 25 diagnosed with ADHD and 33 controls, were recruited through clinics, pediatricians’ offices, and advertising in the community. Participant characteristics are summarized in Table 1. All children in the ADHD group met DSM-IV-TR criteria for ADHD- Combined Type on the Disruptive Behavior Disorder Rating Scale [28] (at least 6 symptoms of inattention and hyperactivity/impulsivity, with parent-teacher consensus on at least one symptom for each domain) and on the Diagnostic Interview Schedule for Children- Fourth Edition [29]. This subtype was chosen because the majority of the reinforcement literature focuses on either the combined or predominantly hyperactive/impulsive type [16,20,25]; because subtype differences were not the focus of the study, we focused on the most prevalent subtype to reduce heterogeneity of the sample. Typical comorbidity patterns with other externalizing disorders were observed (45% Oppositional Defiant Disorder; 31% Conduct Disorder). Control children had less than three symptoms endorsed by parents and teachers on the inattention and hyperactivity/impulsivity domains of the DBD and did not meet DSM-IV criteria for ADHD on the DISC-IV. Exclusion criteria for both groups included IQ ≤ 80 (Wechsler Intelligence Scale for Children –Fourth Edition), diagnosis of pervasive developmental disorder or psychosis, and use of psychiatric medication other than stimulant medication for ADHD. Children currently taking a stimulant medication (n = 15) discontinued stimulant use at least 24 hours before testing (six children in the ADHD group were stimulant naïve, and four had previously used stimulant medications but were not taking them at the time of testing).

**Table 1:** **Participant characteristics**

	**ADHD (n = 25)**	**Control (n = 33)**	***p*** **-value**
Age	10.8 (1.1)	10.9 (1.0)	.84
Sex (M:F)	22:3	27:6	.50
Race/ethnicity (% minority)	16% (n = 4)	15.2% (n = 5)	.99
WISC (FSIQ)	107.9 (11.86)	112.5 (11.5)	.14
ADHD Symptoms (DBD-RS)			
Inattention-Parent	8.1 (1.2)	0.09 (0.4)	<.001
Hyp/Imp-Parent	7.2 (1.7)	0.03 (0.2)	<.001
Inattention-Teacher	6.3 (2.9)	0.09 (0.4)	<.001
Hyp/Imp-Teacher	5.2 (2.5)	0.06 (0.2)	<.001
Cognition- No Reinforcement	−0.89 (1.0)	0.20 (0.55)	<.001
Cognition- Reinforcement	−0.14 (0.77)	0.60 (0.39)	<.001
Baseline Cognition	−0.12 (0.64)	0.09 (0.25)	.11
SR Composite	0.51 (0.62)	−0.39 (0.49)	<.001

Except where noted, values represent the mean (SD). SR composite = mean of standardized parent-reported SR and child-reported BAS; Cognition = mean of standardized SSRT on the SST, percent accuracy on the 1-and 2-back, and CPT hits; DBD-RS = Disruptive Behavior Disorder Rating Scale, values represent the total number of symptoms endorsed on the DBD-RS (i.e., rated as ‘pretty much’ or ‘very much’).

### Procedures

This study was approved by the IRB at the University at Buffalo, and informed consent and assent were obtained before initiation of study procedures. Following a brief telephone screen, families interested in the study attended a diagnostic evaluation session, during which the DISC-IV, DBD, and WISC were administered, and additional parent- and child-report measures were obtained. Children who met eligibility criteria were scheduled for two all-day (7:30 AM- 5:00 PM) lab sessions approximately one week apart, typically within 1-2 months of the diagnostic evaluation. Lab sessions were structured similar to a summer camp and included blocks of cognitive tasks interspersed with recreational activities between testing periods. Task order was counterbalanced across participants but consistent within participants across days. The first visit was a baseline testing day, during which participants could not earn any points for task performance; the second visit was similar, except that tasks were completed under both reinforcement and no-reinforcement conditions, in alternating blocks. During reinforcement blocks, points were earned on a continuous schedule (details below). At the end of the day, children exchanged points for toys and gift cards. Participants received a $10 gift card for participating in the screening process and earned $15-$18 on the baseline day (visit 1) for completing the visit and for appropriate behavior (e.g., sit in your chair, follow directions). Children could earn up to an additional $33 worth of prizes for correct task performance on the reinforcement day only (visit 2).

### Measures

#### Sensitivity to reward

Parental report of child sensitivity to reward and child self-report of behavioral activation were obtained during the diagnostic session. The current study focuses solely on SR/BAS, so information regarding SP/BIS is omitted. The Sensitivity to Punishment and Sensitivity to Reward Questionnaire for Children was adapted from Colder and O’Connor [22]. It is a four-subscale (impulsivity, drive, reward responsivity, and sensitivity to punishment), 33-item measure in which the caregiver must respond from 1 (*strongly disagree*) to 5 (*strongly agree*) to a series of questions tapping their child’s sensitivity to reward (e.g., “Your child generally prefers activities that involved immediate reward.”) and punishment. The SR subscale demonstrated acceptable reliability in the current sample (α = .87).

The child self-report BIS/BAS scales are a downward extension of the adult version of the questionnaire [30], based on Gray’s theory of personality. Children responded on a 0 (*not true*) to 3 (*very true*) Likert scale to 20 items assessing behavioral inhibition and behavioral activation (e.g., “I get really excited when I see an opportunity to get something that I like.”). Child-reported BAS also exhibited acceptable reliability (α = .73).

### Cognitive tasks

Tasks were presented on a Dell CRT computer monitor connected to a response box, and all tasks were programmed in E-prime (Psychology Software Tools, Pittsburgh, PA; code available from the authors). More detailed information for each task can be found in the primary reports [15,26,27].

#### Stop signal task

The Stop Signal Task (SST) is designed to measure the ability to inhibit responding once a prepotent “Go” response has been established [26,31]. The task began with a block of 32 “Go” trials, during which children were instructed to press the right and left buttons of a response box when the arrow on the computer screen pointed right and left, respectively. A 32-trial “Stop” practice block was then completed, in which children were instructed to withhold responding whenever the “Go” stimulus was followed by the stop signal, a 1000 Hz tone presented for 100 ms on 25% of trials. The first “Stop” trial tone began 350 ms after presentation of the “Go” stimulus and adjusted dynamically based on participant response [32]. On the baseline day, participants completed four blocks of 64 trials each (256 trials total; ~13 min duration), consisting of a 500 ms fixation (a white square fixation on black background) period, a 1000 ms stimulus presentation, a 1000 ms response window, and a 500 ms intertrial interval (ITI).

The second day of testing included four 64-trial test blocks that alternated between continuous reinforcement and no reinforcement (~19 min duration), with reinforcement order counterbalanced across participants (same for all tasks). A 1000 ms feedback period was added between the response and ITI to indicate how many points were earned on that trial. Both speed and accuracy were reinforced in order to balance task requirements and prevent slowing of response speed as a strategy for correctly inhibiting. Children earned five points for a fast “Go” response (faster than their “Go” practice mean RT) and two points for a slow “Go” response. Greater points were awarded for a correct “Stop” that was preceded by a fast “Go” response (15 points) than a correct “Stop” that was preceded by a slow “Go” response (six points). During no-reinforcement blocks for all cognitive tasks, participants were told to “try their best”.

Stop Signal Reaction Time (SSRT) was the primary dependent variable for the SST (SSRT = mean “go” RT – mean stop delay).

#### N-Back

The n-back is a visuo-spatial working memory paradigm that progresses in difficulty by increasing the distance between the original stimulus presentation and when that information must be recalled (i.e., load). During the 1-back and 2-back conditions, children were instructed to indicate whether the stimulus was in the same (30% of trials) or a different (70%) location as the stimulus presented one (1-back) or two (2-back) trials previously.

On both testing days, participants completed a 20-trial practice block before completing test blocks of that load condition. Baseline test blocks consisted of 100-trial blocks of each load condition (300 trials; ~15 min duration). Trial structure included a 100 ms stimulus presentation and 2,900 ms response period. On the second day of testing, participants completed 300 no-reinforcement and 300 reinforcement trials (600 trials total; ~ 30 min duration). Load order was counterbalanced across participants, and within each load participants completed four blocks of 50 trials, with blocks alternating between no-reinforcement and reinforcement (reinforcement order counterbalanced across participants). Trials consisted of a 100 ms stimulus presentation, 2000 ms response period, 500 ms feedback period, and a 400 ms fixation period. On reinforcement trials, participants earned five points for correct target hits and two points for correct rejections of non-target stimuli. Only accuracy was reinforced, as percent accuracy was the primary outcome variable on the n-back task.

The 0-back requires only storage of information. The 1-and 2-back require both storage and manipulation of information, taxing the working memory central executive [33]. To focus on working memory, only the 1-and 2-back are included in the present report. Accuracy declined as load increased, but reinforcement and group had similar effects on the 1- and 2-back [15]; therefore, percent accuracy was averaged across the 1-back and 2-back.

#### Continuous performance task

Sustained attention was assessed with an A-X CPT, during which participants were instructed to press a spacebar only when the letter “A” was followed by an “X” (“hits;” 10% of trials) [27,34]. Following the 20-trial practice block, four uninterrupted 100-trial blocks (400 trials; ~11 min duration), each of which contained 10 target A-X pairs (40 targets total), were completed on the baseline day. The reinforcement version consisted of four blocks of 200 trials each, alternating between continuous reinforcement and no reinforcement (800 trials total; ~25 min duration). Trial structure included a 150 ms stimulus presentation, 1000 ms response period, 200 ms feedback period, and 500 ms ITI. As in previous research [27], participants were reinforced for accuracy, rather than speed, and earned 10 points for correct hits and 1 point for correct rejections. Total number of hits (correct responses to target A-X pairs; 80 targets total) was the primary outcome variable.

### Data reduction

#### Cognition composite scores

All cognitive performance variables were averaged across blocks within reinforcement condition. Two participants, one in the ADHD and one in the control group, did not complete the n-back. The data for performance on specific tasks are detailed in task-specific papers [15,26,27]. Here, we aggregate the data for each child across the three paradigms and focus on understanding – rather than evaluating – reinforcement effects on cognitive performance. Composite scores increased reliability of our index of cognition, reduced the number of statistical tests conducted, and enhanced generalizability of results beyond any particular cognitive domain. For the baseline cognition composite, SSRT, n-back percent accuracy, and CPT hits were each standardized, and the average of the three z-scores was computed. Reinforcement and no-reinforcement composites were computed in the same fashion, except that the distribution used for computing z-scores included two scores for each child, one for reinforcement and one for no-reinforcement (separate distributions would have artificially eliminated the critical reinforcement effects of interest). All cognitive composites demonstrated good internal consistency in the current sample; baseline α = .85, no-reinforcement α = .84, reinforcement α = .81.

#### SR composite

A multi-informant composite of parent-reported SR and child-reported BAS was computed to index children’s sensitivity to reinforcement. Each of the three SR subscales on the parent-reported SPSRQ (impulsivity, drive, reward responsivity) and the child-reported BAS scale were standardized and then averaged. The SR composite had acceptable internal consistency for a multi-informant composite (α = .65).

#### Data analytic plan

Data were first screened for outliers and adherence to model assumptions. Diagnostic group differences in baseline cognition and composite SR were examined with t-tests and Cohen’s *d* as the effect size estimate.

Five random effects models were subsequently examined; cognitive composite score was the dependent measure, and the random intercept (i.e., performance during the no-reinforcement condition of the reinforcement task) and main effect of reinforcement were included in all models. Building on our reports for each component of the cognitive composite [15,26,27], Model 1 included the diagnostic group main effect and interaction with reinforcement condition. Diagnostic group was replaced with baseline cognition in Model 2 and SR in Model 3. To determine the degree to which the Diagnostic Group × Reinforcement interaction (Model 1) accounted for variance that was unique versus shared with the Baseline Cognition × Reinforcement interaction (Model 2) and/or the SR × Reinforcement interaction (Model 3), all predictors from Models 1 and 2 were evaluated simultaneously in Model 4, and all predictors from Models 1 and 3 were examined simultaneously in Model 5. A supplemental model was run with all three main effects and interaction terms. Though the logic of this approach is akin to mediation, we did not test our question using a formal mediated moderation procedure because there is no clear temporal precedence among diagnostic group, baseline cognition, and SR.

Effect sizes for Models 1-3 were calculated by the formula outlined by Kreft and de Leeuw [35] and represent the incremental variance explained by the diagnostic group, baseline cognition, and SR’s interactions with reinforcement, respectively, in comparison to a model containing only the main effects. To determine effect sizes in Models 4 and 5, the increment in variance accounted for by the interaction terms of interest were computed by comparing models with and without each of the interaction terms in the full model.

## Results

### Preliminary analyses

As previously reported for this sample, Table 1 shows that groups did not differ on demographic variables or IQ, *p*s > .10; as expected, ADHD children scored significantly higher on parent- and teacher-reported inattention and hyperactivity/impulsivity symptoms, *p*s < .001. Children with ADHD had higher scores on the SR composite measure, *t*(1, 56) = 6.2, *p* < .001, Cohen’s *d* = 1.6 (see Table 1); the group difference on the baseline cognitive composite was moderate in size (Cohen’s *d* = .43) but fell shy of statistical significance, *t*(1, 56) = 1.6, *p* = .11.

### Models 1, 2, and 3 – Independent effects of diagnostic group, baseline cognition, and SR

**Figure 1:**
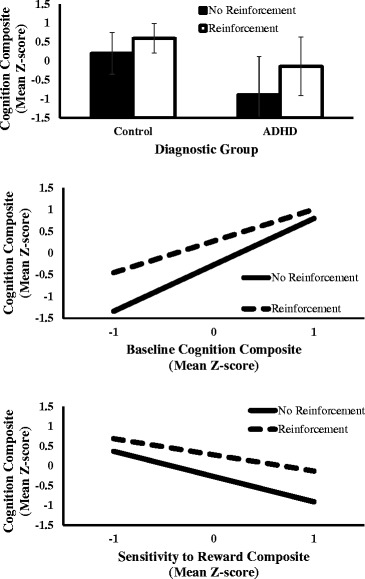
Reinforcement effects on cognition, moderated by diagnostic group, baseline cognition, and sensitivity to reward. Participants completed tasks of inhibitory control (Stop Signal Task), working memory (n-back), and attention (Continuous Performance Task) at baseline (visit 1) and again one week later under alternating reinforcement and no-reinforcement conditions (visit 2). Cognition composites are the average of standardized performance on the three cognitive tasks. The top panel represents the effects of reinforcement on cognition for ADHD and control children. The middle panel represents the effects of reinforcement on cognition across low, average, and high levels of baseline cognition. The bottom panel represents the effects of reinforcement on cognition across low, average, and high levels of Sensitivity to Reward (standardized composite of parent-reported SR and child-reported BAS).

Across models, the main effect of reinforcement condition was robust, *F*s = 100.8-121.1, *p*s < .001. In the diagnostic group-only model (Model 1), the tendency for children with ADHD to exhibit worse cognitive performance than control children, *F*(1, 69.5) = 37.0, *p* < .001, interacted with reinforcement, Diagnostic Group × Reinforcement interaction, *F*(1, 58) = 10.6, *p* = .002 (Table 2 provides parameter estimates and effect sizes for the interaction terms in each model). As shown in Figure 1 (top panel), there was a stronger reinforcement effect on cognition in the ADHD group, *F*(1, 58) = 83.8, *p* < .001, η^2^ = .83, than in the control group, *F*(1, 58) = 30.1, *p* < .001, η^2^ = .57.

**Table 2:** **Parameter estimates and effect sizes for interaction terms predicting cognition**

	**Group × Reinforcement**		**Baseline × Reinforcement**		**Sensitivity to Reward × Reinforcement**	
**Model**	**Coeff (SE)**	**η** ^**2**^	**Coeff (SE)**	**η** ^**2**^	**Coeff (SE)**	**η** ^**2**^
1	0.18 (.05)	.30				
2			−0.34 (.12)	.22		
3					0.23 (.08)	.24
4	0.15 (.05)	.25	−0.27 (.11)	.16		
5	0.13 (.07)	.09			0.11 (.10)	.03

Group refers to diagnostic group (ADHD vs. control); baseline refers to baseline cognition composite = mean of standardized SSRT on the SST, percent accuracy on the 1-and 2-back, and CPT hits; Sensitivity to Reward = mean of standardized parent-reported SR and child-reported BAS.

Empty spaces signify that the interaction term of that column was not tested in that model.

In Model 2, baseline cognition predicted cognitive performance one week later, *F*(1, 69) = 28.4, *p* < .001, and the effect of reinforcement on cognitive performance was qualified by baseline cognition, Baseline Cognition × Reinforcement, *F*(1, 58) = 8.2, *p* = .006. Reinforcement significantly enhanced cognitive performance for those with poor baseline cognition (1 SD below the mean), *F*(1, 58) = 46.0, *p* < .001, η^2^ = .68, but not for those with high baseline cognition (1 SD above the mean), *F*(1, 58) = 2.6, *p* = .11, η^2^ = .06 (Figure 1, middle panel).

Higher SR reliably predicted worse cognitive performance, *F*(1, 68.2) = 21.7, *p* < .001. Reinforcement effects on cognition were moderated by SR, SR × Reinforcement *F*(1, 58) = 8.3, *p* = .005, such that reinforcement improved cognition more strongly for those high in SR, *F*(1, 58) = 65.5, *p* < .001, η^2^ = .78, than those low in SR, *F*(1, 58) = 11.5, *p* = .001, η^2^ = .30 (Figure 1, bottom panel).

### Models 4 and 5 – Diagnostic group and baseline cognition or SR

When diagnostic group status and baseline cognition were included in the model simultaneously, the main effects of group, *F*(1, 72) = 33.3, *p* < .001, and baseline cognition, *F*(1, 72) = 24.9, *p* < .001, remained significant. In terms of the critical interactions with reinforcement condition, both the Diagnostic Group × Reinforcement interaction, *F*(1, 58) = 8.0, *p* = .006, and the Baseline Cognition x Reinforcement interaction remained significant, *F*(1, 58) = 5.7, *p* = .02, and their effect sizes were relatively unchanged (see Table 2).

The diagnostic group and SR model revealed that the main effect of diagnostic group, *F*(1, 69.5) = 14.3, *p* < .001, but not SR, *F*(1, 69.5) = 2.2, *p* = .15, remained significant when both predictors were included in the model. Results suggest significant overlapping variance between diagnostic group and SR, as the Diagnostic Group × Reinforcement interaction became non-significant, *F*(1, 58) = 3.3, *p* = .08, and there was no evidence of the SR × Reinforcement interaction, *F*(1, 58) = 1.3, *p* = .27; effect sizes for each interaction term were markedly attenuated in the presence of the other (see Table 2).

### Supplemental model – Diagnostic group, baseline cognition, and SR

The supplemental model that included the main effects of diagnostic group, baseline cognition, and SR, and each variable’s interaction with reinforcement condition yielded similar results as Models 4 and 5, such that the main effects of baseline cognition, *F*(1, 72) = 24.0, *p* < .001, and diagnostic group remained significant, *F*(1, 72) = 14.2, *p* < .001, but only the Baseline Cognition × Reinforcement interaction remained significant, *F*(1, 58) = 5.4, *p* = .02.

## Discussion

Cognitive and motivational impairments have been central in leading theories of ADHD, and most research to date has understandably focused on evaluating diagnostic group differences in reinforcement effects on cognition. To advance the field, it is critical to examine constructs that may contribute to observed group differences in response to reinforcement. This approach is consistent with the Research Domain Criteria (RDoC) strategy recently adopted by the US National Institute of Mental Health (NIMH) [36,37] which emphasizes processes and dimensions related to psychopathology, rather than diagnostic status per se.

To this end, the current study considered individual differences in baseline cognition and Sensitivity to Reward (SR). Children completed baseline measures of inhibitory control, working memory, and attention during an initial visit and returned a week later to complete the same tasks under reinforcement and no-reinforcement testing blocks. This design provided information about baseline cognitive performance prior to the introduction of a reinforcement condition in which children worked for personally-selected prizes (points were redeemable for a range of prizes ranging in value from $0.50 to $30) in a counterbalanced A-B-A-B design that prevented confounding of reinforcement and time-on-task [25]. We aggregated multiple laboratory measures in the cognitive domain and in parent and child assessments of SR to create composite indices in order to increase reliability and reduce the number of statistical tests.

As in previous research [20], ADHD children had higher SR composite ratings than control children. Even though ADHD children performed worse in each cognitive domain when examined independently [15,26,27], the moderate diagnostic group difference (*d* = .43) in baseline cognition (composite of inhibitory control, working memory, and sustained attention) fell short of statistical significance. This is likely due to the heterogeneity of cognitive deficits in ADHD. In a study of nearly 900 children, Nigg [5] showed that only one-third of children with ADHD show deficits across three cognitive domains. For example, in the current sample, one child with ADHD exhibited poor attention (CPT hits Z = -1.2) but above average inhibitory control (SSRT Z = .29) and working memory (n-back Z = .90), resulting in near-average performance on the cognitive composite.

The choice of domain-specific measures or a cognitive composite depends on the question of interest. If the goal is to demonstrate a group difference or reinforcement effect on a specific aspect of cognition, the more narrow-band measures are a better choice, as broad composites would be less sensitive. However, the main purpose of this paper was to understand the impact of reinforcement on cognition in ADHD more generally. Specifically, we examined the extent to which variation in baseline cognition and/or SR could account for the differential impact of reinforcement on cognition between ADHD and control groups. Relative to examination of specific cognitive domains, we believe the composite approach is advantageous because it allows for more generalized conclusions regarding reinforcement and cognition.

Consistent with our earlier reports for each component of the cognitive composite examined here [15,26,27], the degree to which overall cognition was enhanced by reinforcement was greater for children with ADHD than for control children. In addition, both baseline cognition and SR moderated the effect of reinforcement on cognition in the manner predicted. That is, reinforcement had the greatest effect for those with the lowest baseline cognitive performance and for children who were, according to parent- and self-report, more sensitive to rewards [38,24]. Thus, baseline cognition and SR are both related to the effect of reinforcement on cognitive performance.

Most importantly, to better understand the processes involved in the differential effect of reinforcement in ADHD compared to control children, we examined the degree to which the diagnostic group, baseline cognition, and SR interactions with reinforcement were due to shared variance. Baseline cognition was critical to evaluate from a methodological perspective, as Diagnostic Group × Reinforcement interactions would be of considerably less conceptual interest if simply due to ADHD children having more room for improvement [9,16]. In the present data, the baseline cognition argument was not supported; the Diagnostic Group × Reinforcement and Baseline Cognition × Reinforcement effects both remained significant when evaluated simultaneously.

However, the robust Diagnostic Group × Reinforcement and SR × Reinforcement interactions both became non-significant when tested simultaneously. This pattern suggests that the stronger response to reinforcement among children with ADHD was related to their greater sensitivity to reward. Indeed, some have suggested that reward sensitivity is an endophenotype for ADHD [39]. The overlap between ADHD and SR is broadly consistent with most motivational models of ADHD [16,18]; atypical dopaminergic functioning is a common part of neurobiological explanations of ADHD [18], with research suggesting an exaggerated striatal response to reinforcement in youth with ADHD [19]. Similar regions are thought to underlie SR, and differential striatal activation has been shown to predict behavioral responses to reinforcement [40], suggesting that SR and ADHD share similar neurobiological mechanisms.

More generally, individual differences in SR predict physiological response to reinforcement even among control individuals [24], heightened SR has been implicated in numerous externalizing disorders, particularly substance use [41], and poor responsiveness to appetitive stimuli is predictive of the time course and severity of depression [42]. These findings suggest that SR may be an individual difference variable that cuts across clinical diagnoses, though certain clinical groups (e.g., ADHD) on average tend to be higher in that dimension, leading to greater responses to reinforcement.

Though these data suggest that individual differences in sensitivity to reward are central to understanding reinforcement in ADHD, key replications and extensions are warranted to more fully evaluate this hypothesis. The reliability of the composite SR measure (α = .65) was somewhat low for a trait index, which is likely due to the multi-informant approach used. However, this approach mitigated concerns about a single reporter (parents) driving the relationship between diagnosis and SR. Moreover, the reliability was strong enough that composite SR robustly predicted individual differences in the impact of reinforcement on cognition in the present work. Nevertheless, in the future we plan to extend SR beyond questionnaire measures to behavioral measures of reinforcement, such as progressive ratio reinforcement tasks [43].

Moreover, the present work focused on short periods of alternating positive reinforcement and no reinforcement. Future work should consider negative reinforcement and punishment, as well as evaluate the degree to which reinforcement processes associated with ADHD are static versus dynamic [44]. The generalizability of these results are also limited in that only children with ADHD-Combined Type were included in the study; SR has primarily been linked to externalizing behaviors and impulsivity [22], and its influence on reinforcement effects among predominantly inattentive ADHD individuals has not been investigated.

## Conclusions

In sum, the present work addressed a key issue in reinforcement and ADHD research and theory. Diagnostic group differences in reinforcement effects on cognition have been difficult to interpret because they cannot disentangle differences in reinforcement function from methodological constraints caused by differences in baseline performance [9,16]. Though both baseline cognition and trait SR predicted reinforcement effects on cognition, only SR shared significant variance with diagnostic group. These findings suggest that ADHD children show greater improvement in cognitive task performance with reinforcement not simply because of poorer performance in baseline cognitive functioning, but because of heightened trait sensitivity to reward.
